# Severe, chronic cough caused by pulmonary arteriovenous malformations in a patient with hereditary haemorrhagic telangiectasia: case report

**DOI:** 10.1186/s12890-015-0024-0

**Published:** 2015-03-28

**Authors:** Etienne-Marie Jutant, Philippe Puyo, Mostafa El Hajjam, Sandra Blivet, Emmanuel Houdart, Michel Aubier, Pascal Lacombe, Thierry Chinet

**Affiliations:** APHP, Department of Pulmonology and Thoracic Oncology, Ambroise Paré Hospital, Boulogne, France; Foch Hospital, Department of Thoracic Surgery, Suresnes, France; APHP, Department of Radiology, Ambroise Paré Hospital, Boulogne, France; APHP, Department of Neuroradiology, Lariboisière Hospital, Paris, France; APHP, Department of Pulmonology, Bichat Hospital, Paris, France

**Keywords:** Cough, Hereditary haemorrhagic telangiectasia, Pulmonary arteriovenous malformation

## Abstract

**Background:**

Patients with pulmonary arteriovenous malformations usually complain of dyspnoea upon exertion, fatigue or migraine, or may be asymptomatic. We describe a patient with an unreported manifestation of a pulmonary arteriovenous malformation: a severe chronic cough.

**Case presentation:**

A 51-year old Caucasian non-smoking female police officer presented with a chronic cough. She had been diagnosed with hereditary haemorrhagic telangiectasia in 1992. She complained of a severe, dry cough at the time of the diagnosis and a pulmonary arteriovenous malformation in the upper left lobe as demonstrated by CT of the chest. The fistula was occluded and the cough disappeared rapidly but resumed in 1994. Recanalisation of the fistula led to a new embolisation procedure, and the cough disappeared. Similar episodes occurred in 1998 and 2004, leading to embolisation of a fistula in the right lower lobe and reperfused fistula in the upper left lobe, respectively. The patient was referred to our research team in 2010 because of reappearance of her dry cough that was more pronounced during exercise and exposure to volatile irritants, and absent during the night. Despite extensive investigations, no cause was found other than reperfusion of the fistula in the left upper lobe. The malformation was not accessible to embolisation, leading us to recommend surgical excision of the malformation. A surgeon undertook atypical resection of the left upper lobe in 2012. The cough disappeared immediately after surgery and has not recurred.

**Conclusion:**

Physicians caring for patients with pulmonary arteriovenous malformations should know that a severe, chronic cough can be caused by the malformation. A cough associated with a pulmonary arteriovenous malformation can be treated effectively by embolisation but may resume in cases of reperfusion of the malformation. In our case, the severity of the cough led to surgical excision because embolisation was not possible. The mechanism of action of this cough remains to be determined.

## Background

Pulmonary arteriovenous malformations (PAVMs) are rare. In >80% of cases, PAVMs are associated with hereditary haemorrhagic telangiectasia (HHT), a genetic disease with an autosomal dominant inheritance pattern [1,2]. Acquired PAVMs are associated with hepatic cirrhosis, actinomycosis, trauma and cancers [3]. Other types of PAVMs are presumed to be idiopathic [3].

HHT is a vascular disorder characterised primarily by spontaneous and recurrent epistaxis, telangiectasia on the skin and mucosal membranes, and visceral arteriovenous malformations. Its incidence is ≈ 1 per 10,000 population, and ranges from 1 per 2,000 population to 1 per 40,000 population according to geographical area. Three genes and several loci are associated with HHT. The two most frequent genes are endoglin (*ENG*) on chromosome 9 and of activin-receptor-like-kinase 1 on chromosome 12 [2]. The diagnosis of HHT is based on clinical criteria (Curacao criteria) or the detection of a pathogenic mutation [2]. Approximately 15–50% of patients with HHT have PAVMs [1,2,4]. These PAVMs can be isolated but are usually multiple (on average three fistulae per patient). PAVMs negatively impact the quality of life (QoL) of patients with HHT [5]. They expose patients to severe (sometimes fatal) complications because they cause a right–left anatomical shunt that can let through cruoric, septic or gas emboli [1,3-5]. These paradoxical emboli often affect the central nervous system in the form of strokes, transient ischemic attacks, and brain abscesses, which occur in ≈ 30% of patients [1,3,4,6].

Symptoms of PAVMs are non-specific: dyspnoea upon exertion is the most common sign of PAVMs. Other manifestations of PAVMs include chest pain, fatigue, syncope and migraines. Many patients are asymptomatic. Guidelines recommend screening patients with HHT for the presence of PAVMs [2].

Usual treatment of PAVMs is embolisation (or vaso-occlusion) of the afferent artery if its diameter is >2–3 mm [2,7]. In expert hands, the procedure is low-risk and effective. The main complications of embolisation are: phlebitis; pleural pain; migration of vaso-occlusion material; febrile pleural reactions (sometimes with a delay of several weeks or months). A malformation that has been occluded can be recanalised because of growth of new vessels from pulmonary or systemic circulations [7,8]. Resection of PAVMs is restricted mainly to a few cases of malformations inaccessible to embolisation (e.g., because of their very large size).

We report for the first time a patient with HHT who suffered from severe chronic cough caused by PAVMs that disappeared after embolisation procedures. The cough returned when the occluded PAVM was reperfused, and necessitated excision when embolisation was no longer possible.

## Case presentation

A 51-year-old Caucasian female presented to our specialised HHT Centre with a chronic cough. She was working as a police officer and had never smoked. Her medical history included type-2 diabetes mellitus, cholecystectomy, and HHT. She reported no allergies. She was taking metformin daily.

The diagnosis of HHT was made in 1992 based on the following criteria: spontaneous and recurrent epistaxis; mucocutaneous telangiectasia; family history, and PAVMs. HHT was confirmed in October 2010 with identification of a pathologic mutation on the *ENG* gene.

**Figure 1:**
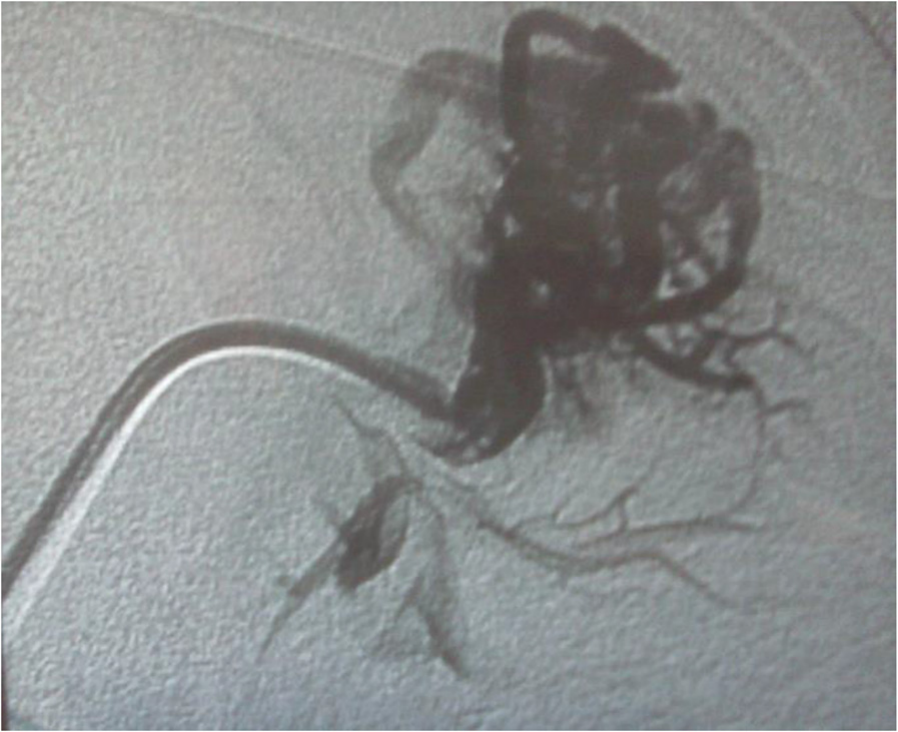
**Pulmonary arteriography in 1992: left apical pulmonary arteriovenous malformation before embolisation.**

**Figure 2:**
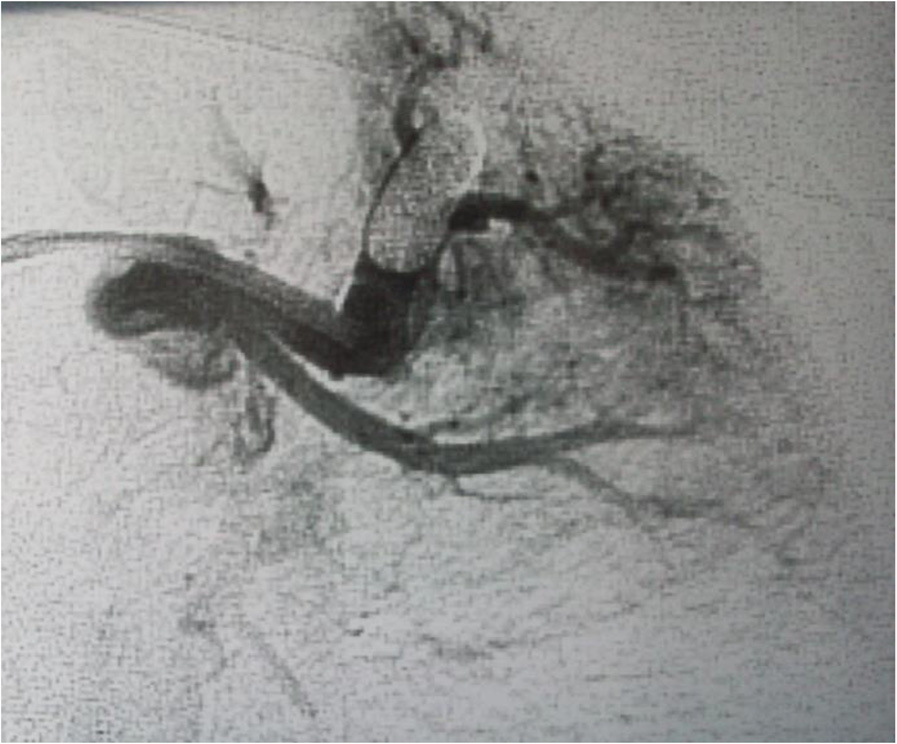
**Pulmonary arteriography in 1992: left apical pulmonary arteriovenous malformation after balloon embolisation.**

PAVMs were discovered in 1992 when the patient sought medical consultation because of a dry, hoarse, hacking cough of several-month duration. CT of the chest showed a fistula in the left upper lobe. The fistula underwent embolisation using a balloon released in a sub-segmental pulmonary artery of the left upper lobe (Figures 1 and 2). The cough disappeared completely 2 days after the procedure.

In 1994, the cough resumed with identical clinical features. New CT of the chest showed an arteriovenous fistula of the right lower lobe close to the diaphragmatic pleura, which was not visible on the previous CT scan. A sub-segmental branch of the right basal pulmonary artery was occluded using two tungsten coils. Embolisation was complicated by an episode of left hemi-anaesthesia that completely resolved within 1 h. CT of the brain revealed two small thalamic hypodensities suggestive of vertebral migration of emboli. The cough disappeared completely 2 days after the procedure.

Pulmonary angiography undertaken 4 months later showed complete exclusion of the left and right embolised fistulae. In 1998, recurrence of the cough led to a new CT of the chest that revealed re-appearance of a small left apical fistula. Pulmonary arteriography confirmed reperfusion of the previously embolised fistula in the left upper lobe by a bronchial artery. Arterial embolisation of this fistula using Histoacryl® glue (B. Braun, Melsungen, Germany) resulted in the cough disappearing within 1 week. A similar episode occurred in 2004 with evidence of reperfusion of the fistula in the left upper lobe by a branch of the left bronchial artery, which was embolised using a mixture of Glubran–Lipiodol® (Guerbet, Aulnay, France). The cough disappeared by the next morning.

**Figure 3:**
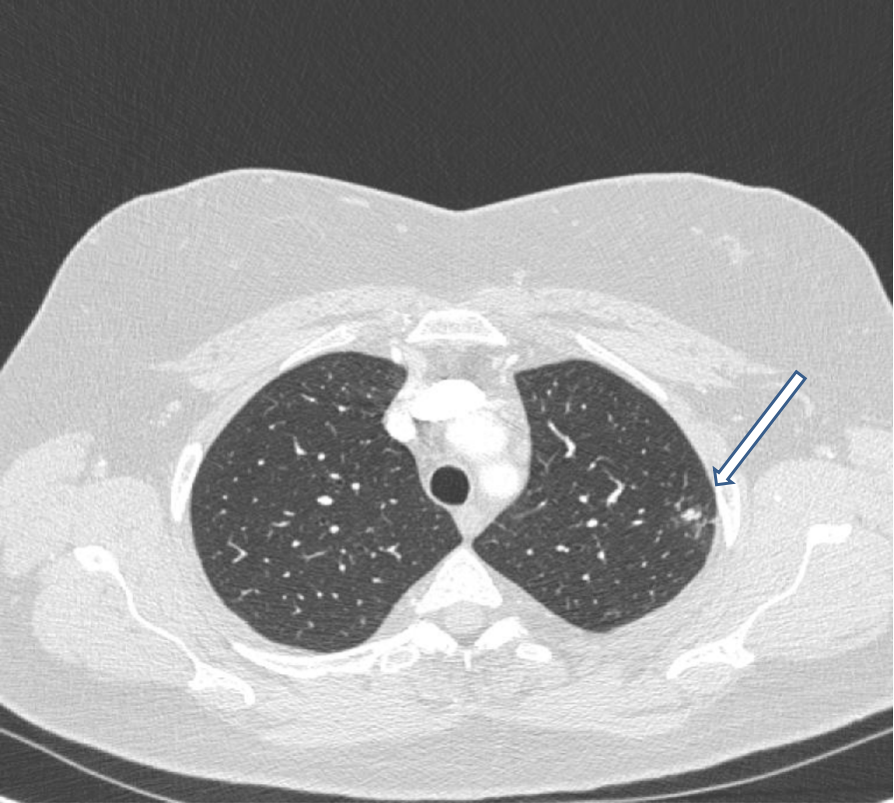
**CT of the chest in 2012 showing a sub-pleural fistula in the left upper lobe (arrow).**

In 2010, the patient was referred to our research team because of the reappearance of a dry cough that was more pronounced during exercise and exposure to volatile irritants, and absent during the night. The cough was so severe that it affected her professional activities, preventing her from contact with the public. Laboratory tests showed a normal blood count and the absence of inflammation. CT of the chest revealed a sub-pleural fistula in the anterior segment of the left upper lobe and a sub-pleural fistula in the right lower lobe (Figure 3).

An iatrogenic cause of the cough was not found. The patient described symptoms of gastro-oesophageal reflux disease. However, endoscopic examination of the upper digestive tract and pH-metry were normal, and oral treatment with a proton pump inhibitor for 8 weeks was ineffective against the cough. The patient reported posterior rhinorrhoea but examination by an ear, nose and throat specialist was normal; symptomatic treatment with an antihistaminic drug and nasal vasoconstrictor cleared up the post-nasal drip but did not affect the cough. Pulmonary function tests were normal; in particular, the investigation for bronchial hyper-responsiveness was negative. Allergy testing was negative. Three-month treatment with inhaled corticosteroids had no effect on the cough. Endoscopic exploration of the bronchi was normal. The cell count and differential of the bronchoalveolar lavage were normal and no infectious organism was retrieved. There were no haemosiderin-laden macrophages. The haemoglobin level had remained >11.0 g/dl during all the years of investigation. It has been reported that premature ventricular contractions (PVCs) can induce a chronic, dry cough, presumably through transient increase in blood flow through pulmonary arteries [9]. However, this patient underwent electrocardiography several times during follow-up yet showed very few PVCs that had no relationships with coughing. In the absence of an obvious cause for the cough, the patient received symptomatic antitussive treatment with codeine for 1 month with no significant effect.

Pulmonary and bronchial arteriography revealed a new simple fistula in the right paracardial segment. In the left lung, pulmonary arteriography showed a lack of peripheral perfusion in the left upper lobe corresponding to the area of the previous embolisations and a small sub-pleural arteriovenous malformation in the left upper lobe fed by a small artery (<1 mm in diameter). Bronchial arteriography visualised a lower left bronchial artery feeding a dense vascular network in the left upper lobe, in the area of the previous embolisations. It was not possible to clearly identify opacification of the left apical fistula. Introduction of a catheter into the left bronchial arteries triggered a cough similar to that experienced by the patient. Embolisation of the right arteriovenous malformation with two coils was not complicated but the cough persisted unchanged after the procedure.

We proposed to surgically remove the left apical fistula given the: (i) negativity of the aetiological investigations of the cough; (ii) absence of effect of medications; (iii) immediate effectiveness of previous embolisation procedures of the fistula in the upper left lobe on the cough; (iv) fact that the cough was triggered by introduction of the catheter into the vessels of the left upper lobe; (v) likelihood of revascularisation of the left apical fistula even if this was not confirmed unequivocally by arteriography; (vi) impossibility of embolisation of the fistula in the upper left lobe; (vii) profound impact of the cough on QoL. The patient was informed of the uncertainties and risks related to the procedure and agreed to undertake the surgical procedure.

**Figure 4:**
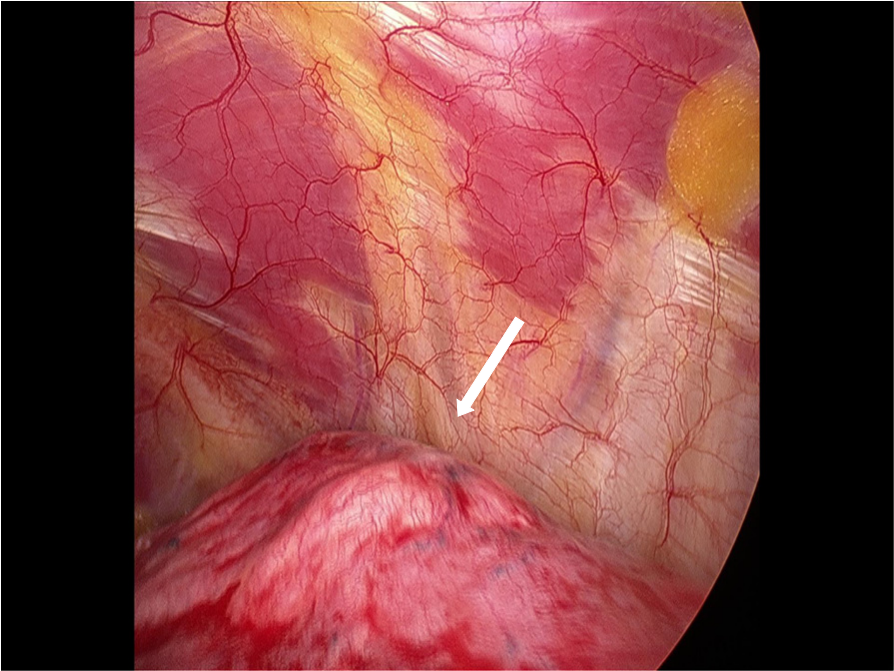
**Sub-pleural vascular malformation of the left upper lobe during surgery (arrow).**

The surgical intervention took place in May 2012. The surgeon found a whitish area surrounded by a vascular network of >1 cm on the surface of the left upper lobe (Figure 4). He carried out atypical resection of the left upper lobe. Pathological examination of the surgical specimen revealed a nodular lesion of ≈ 1.5 cm in contact with thickened fibrotic pleura. This lesion was constituted microscopically by abnormal arterial and venous vessels, bronchioles and alveoli. There were numerous focal fibroelastic lesions in the pulmonary parenchyma. Arteries exhibited thickened walls with fibrosis of the media, reduction in calibre, sometimes complete occlusion and, in places, reperfusion. Veins were also occluded in places. Upon awakening, the patient was no longer coughing. The procedure had no complications except for a hiccup that completely disappeared after 2 months.

Twenty-six months after the surgical procedure, the cough has not recurred and the patient has no functional complaints. She has resumed her professional activities fully.

## Discussion

This case study demonstrates that a severe, chronic cough can be caused by a PAVM. In our patient, evidence led us to consider that the arteriovenous fistula in the left upper lobe was responsible for the chronic cough. Disappearance of the cough after previous embolisation procedures and immediately after surgical excision confirmed this hypothesis. The mechanism by which an arteriovenous fistula in the lung can cause coughing is not clear. One possibility could be irritation of the pleura due to the sub-pleural location of the fistula in the left upper lobe, as well as the first fistula in the right lower lobe. Alternatively, we observed that introduction of a catheter into the left bronchial arteries triggered a cough similar to that experienced by the patient. This phenomenon could have been due to irritation of the arterial wall or modification of blood flux into the artery and, therefore, the fistula. From 1992, reappearance of the cough was associated with demonstration of reperfusion of the embolised fistula in the left upper lobe, which may, therefore, relate the cough to blood flow through the fistula. The indication for surgery was made given the impact of the cough on the patient’s QoL and impossibility of carrying out a new embolisation procedure.

In patients with HHT, PAVMs may grow over time [1-3]. However, the exact biological mechanisms of how mutations in endoglin and acvrl1 lead to the growth of arteriovenous malformations are not known, nor why only a fraction of PAVMs increase in size over time in adults.

## Conclusion

PAVMs can cause a severe, incapacitating, chronic cough. The cough associated with a PAVM can be treated effectively by embolisation but may resume in cases of reperfusion of the malformation. In our case, the severity of the cough led to surgical excision because embolisation was not possible. The mechanism of this cough has yet to be determined.

## Consent

Written informed consent was obtained from the patient for publication of this case report and accompanying images. A copy of the written consent is available for review by the Editor of this journal.

## Abbreviations

ENG: Endoglin
HHT: Hereditary haemorrhagic telangiectasia
PAVM: Pulmonary arteriovenous malformations
QoL: Quality of life
